# Prognostic impact of pretreatment T790M mutation on outcomes for patients with resected, *EGFR*-mutated, non-small cell lung cancer

**DOI:** 10.1186/s12885-022-09869-7

**Published:** 2022-07-15

**Authors:** Yoshiya Matsumoto, Tomoya Kawaguchi, Masaru Watanabe, Shun-ichi Isa, Masahiko Ando, Akihiro Tamiya, Akihito Kubo, Chiyoe Kitagawa, Naoki Yoshimoto, Yasuhiro Koh

**Affiliations:** 1 Department of Respiratory Medicine, Graduate School of Medicine, Osaka Metropolitan University, Osaka, Japan; 2 Internal Medicine III, Wakayama Medical University, Wakayama, Japan; 3 Clinical Research Center, National Hospital Organization Kinki-chuo Chest Medical Center, Sakai, Japan; 4 Advanced Medicine and Clinical Research, Nagoya University Hospital, Nagoya, Japan; 5 Internal Medicine, National Hospital Organization Kinki-chuo Chest Medical Center, Sakai, Japan; 6 Division of Respiratory Medicine and Allergology, Department of Internal Medicine, Aichi Medical University School of Medicine, Nagakute, Japan; 7 Medical Oncology and Respiratory Medicine, Nagoya Medical Center, Nagoya, Japan; 8 Respiratory Medicine, Ishikiriseiki Hospital, Higashiosaka, Japan; 9 Center for Biomedical Sciences, CIMS, Wakayama Medical University, 811-1 Kimiidera, Wakayama-shi, Wakayama, 641-8509 Japan

**Keywords:** Non-small cell lung cancer, EGFR mutation, Pretreatment T790M, Resection, Recurrence-free survival

## Abstract

**Background:**

Many previous studies have demonstrated that minor-frequency pretreatment T790M mutation (preT790M) could be detected by ultrasensitive methods in a considerable number of treatment-naïve, epidermal growth factor receptor (*EGFR*)-mutated, non-small cell lung cancer (NSCLC) cases. However, the impact of preT790M in resected cases on prognosis remains unclear.

**Methods:**

We previously reported that preT790M could be detected in 298 (79.9%) of 373 surgically resected, *EGFR*-mutated NSCLC patients. Therefore, we investigated the impact of preT790M on recurrence-free survival (RFS) and overall survival (OS) in this cohort by multivariate analysis. All patients were enrolled from July 2012 to December 2013, with follow-up until November 30, 2017.

**Results:**

The median follow-up time was 48.6 months. Using a cutoff value of the median preT790M allele frequency, the high-preT790M group (*n* = 151) had significantly shorter RFS (hazard ratio [HR] = 1.51, 95% confidence interval [CI]: 1.01–2.25, *P* = 0.045) and a tendency for a shorter OS (HR = 1.87, 95% CI: 0.99–3.55, *P* = 0.055) than the low-preT790M group (*n* = 222). On multivariate analysis, higher preT790M was independently associated with shorter RFS (high vs low, HR = 1.56, 95% CI: 1.03–2.36, *P* = 0.035), irrespective of advanced stage, older age, and male sex, and was also associated with shorter OS (high vs low, HR = 2.16, 95% CI: 1.11–4.20, *P* = 0.024) irrespective of advanced stage, older age, *EGFR* mutation subtype, and history of adjuvant chemotherapy.

**Conclusions:**

Minor-frequency, especially high-abundance of, preT790M was an independent factor associated with a poor prognosis in patients with surgically resected, *EGFR*-mutated NSCLC.

**Supplementary Information:**

The online version contains supplementary material available at 10.1186/s12885-022-09869-7.

## Background

Over the past decade, the treatment paradigm for advanced non-small cell lung cancer (NSCLC) has evolved dramatically due to the detection of “druggable” gene alterations and the development of molecular-targeted therapies [1]. Mutations in the epidermal growth factor receptor (*EGFR*) gene, such as exon 19 deletions (19del) and Leu858Arg point mutations in exon 21 (L858R), are among the most common driver oncogenes in NSCLC. EGFR tyrosine kinase inhibitors (TKIs) are the recommended first-line treatment for advanced NSCLC patients with *EGFR*-activating mutations and improve survival significantly in those patients. However, most patients received EGFR-TKI therapies eventually develop resistance. The most common mechanism of acquired resistance to first-generation or second-generation EGFR-TKIs, such as gefitinib, erlotinib, and afatinib, is secondary *EGFR* T790M mutation, which is observed in 50–60% of acquired resistance cases [2, 3]. Osimertinib is a third-generation EGFR-TKI that selectively inhibits both *EGFR*-activating and T790M mutations. Based on the remarkable results of the AURA and FLAURA trials, osimertinib has been approved for the treatment of advanced NSCLC patients with *EGFR* mutations as first-line treatment and *EGFR* T790M-positive patients who had disease progression after prior-line EGFR-TKI treatment [4, 5].

The origin of EGFR-TKI resistance due to T790M mutation is not yet well understood. One of the hypotheses was that a T790M clone as a minor de novo clone preexists in treatment-naïve tumors, and the pretreatment/de novo T790M (preT790M) clone is selected and enriched by exposure to EGFR-TKIs [6, 7]. Actually, earlier studies indicated the presence of minor-frequency T790M in pretreatment tumor samples in a small cohort, but detection rates varied from 2 to 79%, depending on the mutation detection methods and their sensitivities [7–13]. Therefore, we previously conducted a study aimed to accurately detect preT790M and clarify the prevalence of preT790M in a larger cohort that was an *EGFR*-mutant subset within the Japan Molecular Epidemiology (JME) study cohort, and we reported that preT790M was detected in 298 (79.9%) of 373 *EGFR*-mutated NSCLC patients using the ultra-sensitive droplet digital polymerase chain reaction (ddPCR), the analytical sensitivity of which was approximately 0.001% [14]. Recent studies using ddPCR also reported that the detection rates of minor-frequency preT790M were 7.9–70.6% [15–19].

In a recent report, the phase 3 randomized ADAURA trial, which investigated the efficacy and safety of osimertinib as adjuvant treatment compared with placebo after adjuvant chemotherapy in patients with completely resected stage IB to IIIA *EGFR*-mutated NSCLC, showed significant improvement in disease-free survival (DFS) in the adjuvant osimertinib arm [20]. Based on the result, osimertinib as adjuvant therapy for NSCLC patients with *EGFR* mutations has been approved in the United States, China, and the European Union. However, there remain issues with adjuvant EGFR-TKI therapy, such as unclearness of overall survival (OS) benefit, the optimal administration period, cost, and adverse events. Therefore, development of biomarkers that identify high-risk populations for postoperative recurrence are needed to avoid unnecessary treatment. In advanced stage settings, previous researches demonstrated that preT790M was related to poor efficacy or shorter progression-free survival with the early-generation EGFR-TKI treatment [8–10, 12, 13, 16, 18, 19, 21, 22]. Moreover, several studies showed that a higher mutant-allele frequency (MAF) of preT790M might have greater impact on the efficacy of EGFR-TKIs than the presence of preT790M [12, 13, 18, 19]. These data suggest the negative predictive value of preT790M abundance for the efficacy of early-generation EGFR-TKIs in patients with advanced stage *EGFR-*mutated NSCLC. Therefore, preT790M could be a potential biomarker candidate to identify patients who may benefit from adjuvant osimertinib treatment. However, the clinical significance or prognostic implications of minor-frequency preT790M in patients with early-stage *EGFR-*mutated NSCLC who had undergone surgical resection have not yet been determined.

The JME study is a prospective, multicenter, molecular epidemiology study collecting samples from 876 surgically resected NSCLC cases and examining the somatic mutations to tackle associations between driver oncogenes and smoking and other environmental factors. Molecular profiling of that cohort as the primary endpoint of the JME study has been previously reported [23]. The secondary endpoints of the study were recurrence-free survival (RFS) and OS analyses (UMIN 000008177). Thus, the follow-up data and clinical outcomes were collected prospectively with the intent to investigate the impact of somatic mutations on RFS and OS for resected NSCLC. In this report, the follow-up data and clinical outcomes, focused on the *EGFR* mutant cohort of the JME study, are presented, and the impact of preT790M in patients with surgically resected *EGFR*-mutated NSCLC on RFS and OS is elucidated.

## Methods

### Patients

Eligible subjects from the JME study were pathologically diagnosed NSCLC patients with clinical stage I to IIIB disease (TNM classification version 7) who had undergone surgery for therapeutic purposes. Full details of the study design have been published previously [23]. All patients were enrolled from July 2012 to December 2013. Somatic mutations were analyzed by multiplex-targeted deep sequencing, and mutations in *EGFR* were also confirmed by PCR methods by an independent clinical laboratory (SRL, Tokyo, Japan). As a result, 373 samples with an *EGFR*-activating mutation based on the Cycleave PCR method were analyzed by ddPCR in the current JME substudy. This study was approved by the Institutional Review Board of the National Hospital Organization of Japan. All patients provided written, informed consent before surgery. The study was conducted in accordance with the Declaration of Helsinki.

### Detection of *EGFR* T790M mutation by droplet digital PCR

The ddPCR was carried out with a RainDrop Digital PCR system (Bio-Rad, Hercules, CA, USA), and the details of this procedure have been published previously [14]. Briefly, the duplex assay is based on the concurrent amplification of wildtype and specific mutant sequences in picoliter-sized compartmentalized liquid droplets and measurement of the terminal fluorescence signal from each droplet by flow cytometric techniques.

### Statistical analysis

Clinical data, including age, sex, smoking history, pathological stage, history of adjuvant chemotherapy, *EGFR* mutation subtype, and study findings including preT790M were used for the analysis of the current JME substudy. Fisher’s exact test was carried out for comparison of categorical data. The Kaplan–Meier method was used to estimate the survival curves for RFS and OS. Log-rank tests were used to compare the survival curves among the patients by preT790M status. RFS was defined as the period from the date of operation to the date of confirmed recurrence from any cause. Patients that were alive on the date of the last follow-up were censored at the time. All *P*-values were according to a two-sided hypothesis, and a *P*-value < 0.05 was considered significant. A Cox proportional hazards model was used to evaluate the impact of preT790M on RFS and OS. Statistical analysis was performed using IBM SPSS software (version 25).

## Results

### Patient characteristics

In the current JME substudy, 373 *EGFR*-mutated NSCLC samples were obtained, and all samples could be analyzed for preT790M by ultra-sensitive ddPCR. As reported previously, preT790M was detected in 298 (79.9%) of 373 *EGFR*-mutated NSCLC patients using ddPCR, of which the analytical sensitivity was approximately 0.001%. The preT790M-MAF ranged from 0.009 to 26.9% (median MAF 0.044%), and most tumors had preT790M-MAF < 0.1% [14].

All 373 patients’ clinical and prognostic data were collected prospectively. The data cutoff date for the JME study was November 30, 2017, and the median follow-up time in this study was 48.6 months. The characteristics of the patients are shown in Table 1. In the current analysis, the median age was 69 years (range 30–92 years), 182 patients (48.8%) were 70 years old or older, 276 (74.0%) were female, 361 (96.8%) were diagnosed with adenocarcinoma, and 93 (24.9%) had a smoking history. The number of patients by pathological stage was 219 patients (56.7%) in stage IA, 71 (19.0%) in stage IB, 43 (11.5%) in stage II, and 40 (10.7%) in stage III-IV. A total of 120 patients (32.2%) received the adjuvant chemotherapy, and of these, 2 patients with stage II and 5 patients with stage III-IV received gefitinib as adjuvant chemotherapy. Regarding *EGFR* mutation status, 155 patients (41.6%) had 19del, 199 (53.4%) had L858R, and 19 (5.1%) had uncommon mutations.

**Table 1:** Baseline characteristics of patients according to pretreatment T790M status

Characteristics	Number of patients, n (%)						*P*
	All (*n* = 373)		Pretreatment T790M				
			High (n = 151, 40.5%)		Low (n = 222, 59.5%)		
Age							0.67
Median (range)	69	(30–92)	69	(37–88)	69	(30–92)	
< 70	191	(51.2)	75	(49.7)	116	(52.3)	
≥ 70	182	(48.8)	76	(50.3)	106	(47.7)	
Gender							0.47
Male	97	(26.0)	36	(23.8)	61	(27.5)	
Female	276	(74.0)	115	(76.2)	161	(72.5)	
Smoking							0.0050
Never smoker	280	(75.1)	125	(82.8)	155	(69.8)	
Smoker	93	(24.9)	26	(17.2)	67	(30.2)	
Histology							0.24
Adenocarcinoma	361	(96.8)	144	(95.4)	217	(97.7)	
Other	12	(3.2)	7	(4.6)	5	(2.3)	
Pathological Stage (7th)							0.42
IA	219	(58.7)	84	(55.6)	135	(60.8)	
IB-II	114	(30.6)	52	(34.4)	62	(27.9)	
III-IV	40	(10.7)	15	(9.9)	25	(11.3)	
*EGFR* Mt Status							0.92
Exon21 L858R	199	(53.4)	82	(54.3)	117	(52.7)	
Exon19 deletion	155	(41.6)	62	(41.1)	93	(41.9)	
Uncommon	19	(5.1)	7	(4.6)	12	(5.4)	
Adjuvant Chemotherapy						(69.8)	0.37
No	253	(67.8)	98	(64.9)	155	(30.2)	
Yes	120	(32.2)	53	(35.1)	67		
UFT	73		32		41		
Platinum doublet	34		18		16		
EGFR-TKI	7		1		6		
Other	6		2		4		
*TP53* Mt Status						(78.4)	0.077
Wild-type	304	(81.5)	130	(86.1)	174		
Mutant	69	(18.5)	21	(13.9)	48	(21.6)	
Co-existing except *TP53* Mt						(89.2)	0.52
No	329	(88.2)	131	(86.8)	198	(10.8)	
Yes	44	(11.8)	20	(13.2)	24		

*EGFR* Epidermal growth factor receptor, *Mt* Mutation, *UFT* Tegafur-uracil, *TKI* Tyrosine kinase inhibitor, *TP53* Tumor protein P53

When tumor samples were classified as having low or high levels of preT790M, using MAF of 0.045% as the cutoff based on the median MAF of 0.044%, that is, low-preT790M was defined as consisting of preT790M-MAF < 0.045% plus preT790M-negative, and high-preT790M was defined as preT790M-MAF ≥0.045%, the low group had 222 patients (59.5%), and the high group had 151 patients (40.5%). According to preT790M status, there were no significant differences in preT790M status by age, sex, tumor stage, *EGFR* mutation status, or history of adjuvant chemotherapy. However, there were significantly more never smokers among patients with high-preT790M than among those with low-preT790M (Table 1).

### Impact of pretreatment T790M on RFS

**Fig. 1:**
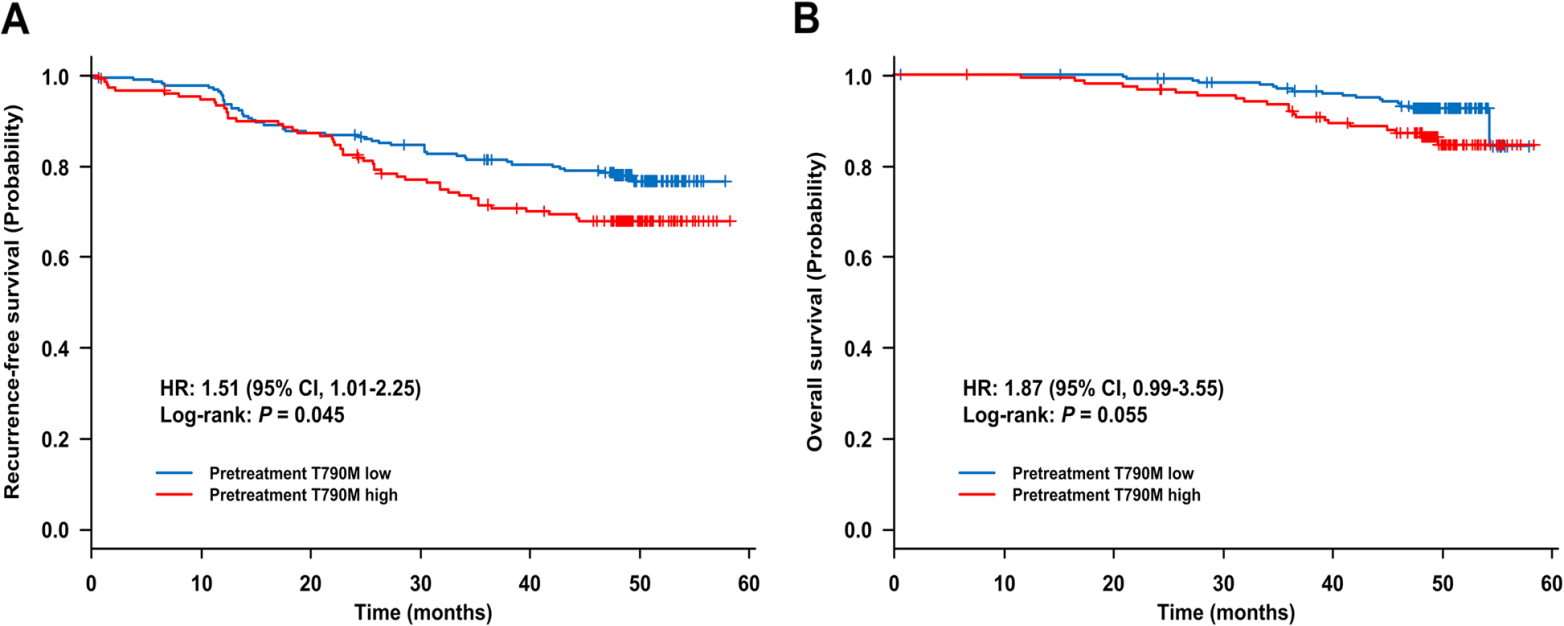
Kaplan-Meier curves for recurrence-free survival (A) and overall survival (B) according to pretreatment T790M status (high vs low). Plus signs denote censoring

First, RFS was analyzed according to preT790M status. RFS tended to be shorter in patients with preT790M than in those without preT790M (hazard ratio [HR] = 1.76, 95% confidence interval [CI]: 0.98–3.15, *P* = 0.056) (Supplementary Fig. S1A), whereas RFS was significantly shorter in patients with high-preT790M than in those with low-preT790M (HR = 1.51, 95% CI: 1.01–2.25, *P* = 0.045) (Fig. 1A). On univariate analysis, age (≥70 years), pathological stage (III-IV > IB-II > IA), and adjuvant chemotherapy (yes) were factors related to shorter RFS. On the other hand, sex, smoking history, and *EGFR* mutation status did not affect RFS (Table 2). During this observational period, 96 RFS events were occurred.

**Table 2:** Prognostic factors for recurrence-free survival (RFS): univariate and multivariate analyses

Factor	univariate			multivariate		
	HR	(95% CI)	*P*	HR	(95% CI)	*P*
Pretreatment T790M (ref = Low)						
High	1.51	(1.01–2.25)	.045	1.56	(1.03–2.36)	.035
Age (ref = < 70)						
≥ 70	1.85	(1.23–2.79)	<.01	1.55	(0.99–2.43)	.056
Gender (ref = Female)						
Male	1.16	(0.75–1.81)	.50	1.93	(1.06–3.52)	.032
Smoking (ref = Never smoker)						
Smoker	1.16	(0.75–1.82)	.50	0.87	(0.48–1.59)	.66
*EGFR* Mt (ref = Exon21 L858R)						
Exon19 deletion	1.03	(0.68–1.55)	.90	1.02	(0.66–1.57)	.93
Uncommon	1.03	(0.41–2.58)	.95	0.53	(0.20–1.40)	.20
Pathological Stage (7th) (ref = IA)						
IB-II	6.05	(3.51–10.42)	<.00001	6.30	(3.46–11.47)	<.00001
III-IV	19.43	(10.80–34.96)	<.00001	25.46	(13.11–49.44)	<.00001
Adjuvant Chemotherapy (ref = No)						
Yes	2.19	(1.47–3.27)	<.001	0.87	(0.55–1.38)	.56

**Fig. 2:**
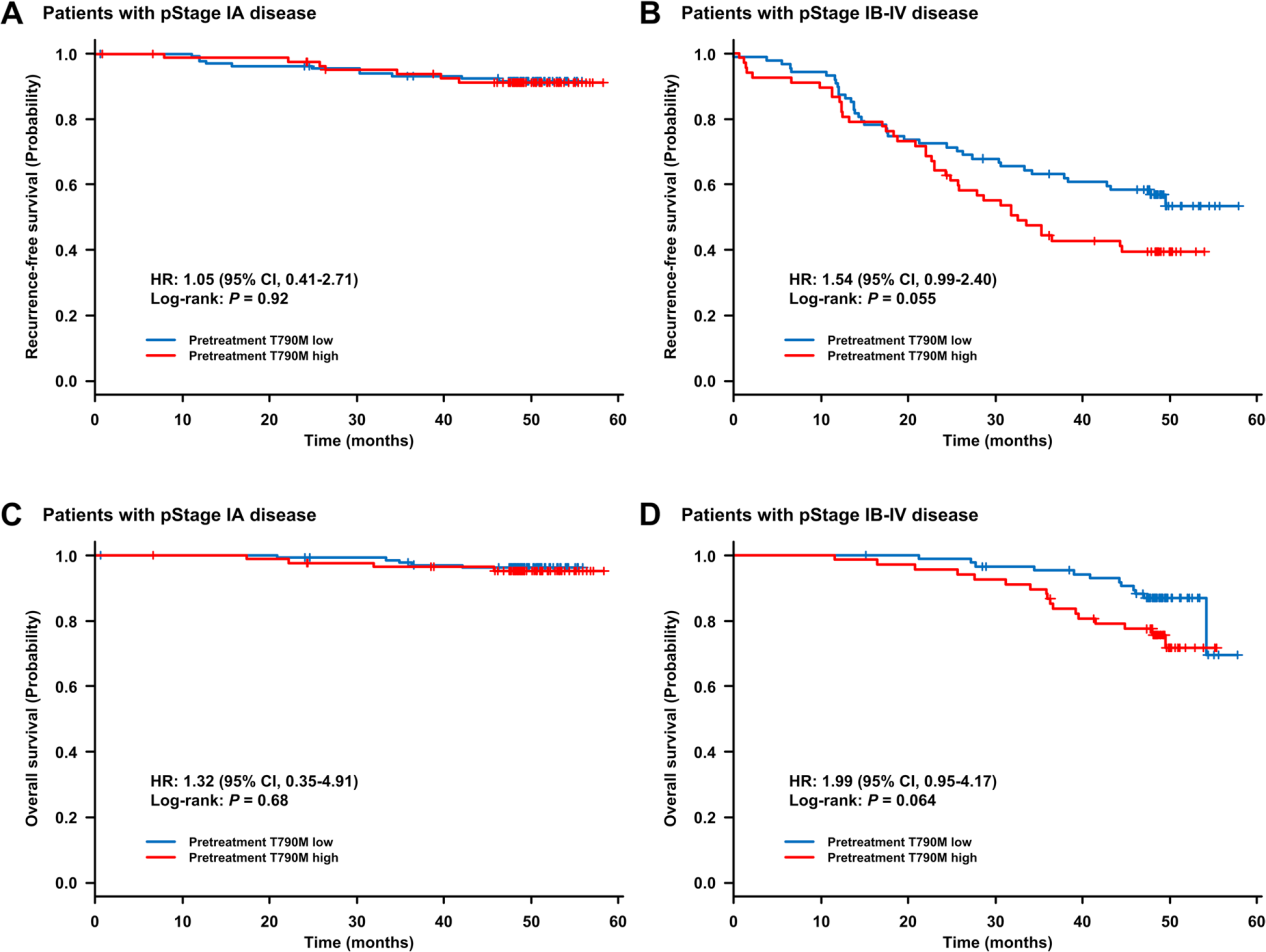
Kaplan-Meier curves for recurrence-free survival (**A**, **B**) and overall survival (**C**, **D**) according to pretreatment T790M status (high vs low). (**A**) and (**C**) for the population with pathological stage IA NSCLC. (**B**) and (**D**) for the population with pathological stage IB-IV NSCLC. Plus signs denote censoring

Multivariate analysis demonstrated that higher preT790M-MAF (high vs low, HR = 1.56, 95% CI: 1.03–2.36, *P* = 0.035), male sex (male vs female, HR = 1.93, 95% CI: 1.06–3.52, *P* = 0.032), and advanced pathological stage (IB-II vs IA, HR = 6.30, 95% CI: 3.46–11.47, *P* < 0.00001; III-IV vs IA, HR = 25.46, 95% CI: 13.11–49.44, *P* < 0.00001) were significantly associated with shorter RFS (Table 2). Older age tended to be related to shorter RFS (≥70 vs < 70 years, HR = 1.55, 95% CI: 0.99–2.43, *P* = 0.056). According to pathological stage, RFS was shorter in patients with high-preT790M than in those with low-preT790M in stage IB-IV, but there was no significant difference in RFS regardless of preT790M status in stage IA (Fig. 2A-B and Supplementary Fig. S2).

### Impact of pretreatment T790M on OS

Second, OS was analyzed according to preT790M status. There was no significant difference in OS between patients with and without preT790M (HR = 1.32, 95% CI: 0.53–3.27, *P* = 0.55) (Supplementary Fig. S1B), but OS tended to be shorter in patients with high-preT790M than in those with low-preT790M (HR = 1.87, 95% CI: 0.99–3.55, *P* = 0.055) (Fig. 1B). On univariate analysis, age (≥70 years), uncommon *EGFR* mutation, and pathological stage (III-IV > IB-II > IA) were factors related to shorter OS. On the other hand, sex, smoking history, and adjuvant chemotherapy did not affect OS (Table 3). During this observational period, 38 OS events were occurred.

**Table 3:** Prognostic factors for overall survival (OS): univariate and multivariate analyses

Factor	univariate			multivariate		
	HR	(95% CI)	*P*	HR	(95% CI)	*P*
Pretreatment T790M (ref = Low)						
High	1.87	(0.99–3.55)	.055	2.16	(1.11–4.20)	.024
Age (ref = < 70)						
≥ 70	2.13	(1.09–4.17)	.028	1.79	(0.87–3.69)	.12
Gender (ref = Female)						
Male	1.12	(0.56–2.26)	.75	1.17	(0.40–3.44)	.77
Smoking (ref = Never smoker)						
Smoker	1.34	(0.68–2.66)	.40	1.25	(0.43–3.61)	.69
*EGFR* Mt (ref = Exon21 L858R)						
Exon19 deletion	1.56	(0.79–3.10)	.20	1.80	(0.88–3.67)	.11
Uncommon	3.54	(1.28–9.77)	.015	3.33	(1.13–9.83)	.029
Pathological Stage (7th) (ref = IA)						
IB-II	3.51	(1.55–7.95)	<.01	4.30	(1.77–10.40)	.001
III-IV	9.68	(4.14–22.67)	<.00001	12.10	(4.83–30.29)	<.00001
Adjuvant Chemotherapy (ref = No)						
Yes	1.07	(0.55–2.09)	.85	0.47	(0.22–1.01)	.053

Multivariate analysis demonstrated that higher preT790M-MAF (high vs low, HR = 2.16, 95% CI: 1.11–4.20, *P* = 0.024), uncommon *EGFR* mutation (vs L858R, HR = 3.33, 95% CI: 1.13–9.83, *P* = 0.029), and advanced pathological stage (IB-II vs IA, HR = 4.30, 95% CI: 1.77–10.40, *P* = 0.001; III-IV vs IA, HR = 12.10, 95% CI: 4.83–30.29, *P* < 0.00001) were significantly associated with shorter OS (Table 3). Older age (≥70 vs < 70 years, HR = 1.79, 95% CI: 0.87–3.69, *P* = 0.12) and 19del (vs L858R, HR = 1.80, 95% CI: 0.88–3.67, *P* = 0.11) tended to have shorter OS, and adjuvant chemotherapy tended to be related to longer OS (HR = 0.47, 95% CI: 0.22–1.01, *P* = 0.053). According to pathological stage, OS was shorter in patients with high-preT790M than in those with low-preT790M in stage IB-IV, but there was no significant difference in OS regardless of preT790M status in stage IA (Fig. 2C-D and Supplementary Fig. S3).

During this observational period, 92 postoperative recurrence events were recorded. According to the preT790M status, 44 (29.1%) and 48 (21.6%) recurrences were observed in the high- and low-preT790M groups, respectively. Post-recurrence treatments are shown in Table 4.

**Table 4:** Postoperative recurrence events and post-recurrence treatment

	Number of patients, n (%)						*P*
	All(n = 373)		Pretreatment T790M				
			High(n = 151)		Low(n = 222)		
Recurrence events	92	(24.7)	44	(29.1)	48	(21.6)	0.11
Locoregional	45	(12.1)	21	(13.9)	24	(10.8)	0.42
Distant	58	(15.5)	28	(18.5)	30	(13.5)	0.19
Post-recurrence treatment							
Local therapy							
Radiation therapy	24		13		11		
Surgery	4		1		3		
Systemic therapy							
Chemotherapy	9		5		4		
EGFR-TKI	53		25		28		
Best Supportive Care	5		1		4		

## Discussion

Before the current study, many studies reported the predictive impact of minor-frequency preT790M on the efficacy of initial EGFR-TKI treatment or on the prognosis, mainly in advanced stage settings. However, there were few reports regarding the clinical significance of preT790M in patients with resected NSCLC. In this prospective exploratory analysis, minor-frequency preT790M in the resected *EGFR-*mutated NSCLC samples was shown to potentially affect RFS and OS.

In previous analyses of prognostic factors for resected *EGFR*-mutated NSCLC, older age, male sex, advanced stage, and smoker were shown to be independent factors associated with a poor prognosis (RFS or OS) in many studies, although the covariates evaluated differed depending on the studies [24–34]. In the current study, multivariate analysis, which considered these previous reports, demonstrated that male sex and advanced pathological stage were correlated with shorter RFS, and older age tended to be associated with shorter RFS. Pathological stage was also correlated with shorter OS, and in fact, pathological stage was the most important factor affecting RFS and OS. There were many reports showing that *EGFR* 19del was associated with worse RFS or OS than L858R [26, 28, 33], but some studies reported that 19del had better RFS or OS than L858R [30, 35], and others reported that *EGFR* mutation subtype has no prognostic impact [25]. Therefore, whether the *EGFR* mutation subtype has an impact on prognosis remained controversial. On multivariate analysis in the current study, there was no significant difference in RFS by *EGFR* mutation subtype, but 19del tended to have worse OS, and uncommon mutations had significantly worse OS than L858R.

Let us consider the impact of preT790M as a prognostic factor for survival. In the current study using ultra-sensitive ddPCR, the overall detection rate of preT790M was 79.9%, and the T790M-MAF ranged from 0.009 to 26.9% (median MAF 0.044%). Several previous studies indicated that higher MAF of preT790M might have a greater impact on the efficacy of EGFR-TKIs than the presence of preT790M [12, 13, 18, 19]. Therefore, when tumor samples were classified into two groups based on the abundance of T790M-MAF, multivariate analysis demonstrated that high-preT790M was the independent factor related to a poor prognosis (RFS), irrespective of patient background, including pathological stage, age, and sex. A previous retrospective study by Tatematsu et al., which analyzed the incidence of minor-frequency preT790M using competitive allele-specific PCR in 153 surgically resected *EGFR*-mutated lung adenocarcinoma tissues, the incidence of preT790M was 29.4%, and T790M-MAF ranged from 0.13 to 2.65% (median MAF 0.20%) [36]. However, in their study, no significant impact of preT790M on RFS was shown. A previous analysis demonstrated that the impact of T790M shifts according to the cutoff level of T790M-MAF [13]. Therefore, differences in analytical sensitivity, the detection rate of preT790M, sample size, and population grouping might result in the differences in the impact of preT790M between their study and the present one. Furthermore, disease stage was the most crucial factor affecting prognosis [24–29, 31–34]. Therefore, it might be important to consider pathological stage in the analysis of clinical significance of minor-frequency preT790M, although the association of preT790M status with patient characteristics was not reported in their study. In fact, the present study showed that higher-preT790M affected RFS in stage IB or more advanced disease, but it seemed unlikely in stage IA. On the other hand, Gao et al. analyzed clinical outcomes of coexisting T790M in a surgically resected, *EGFR-*mutated NSCLC cohort using the Amplification Refractory Mutation System, of which the analytical sensitivity was known to be generally 1% [37]. Their study also demonstrated that RFS of patients with coexisting *EGFR* T790M was significantly shorter than of those without T790M mutations, and according to the stage, this tendency was observed not only in stage IB-IIIA, but also in stage IA. Greater T790M-MAF, which could be detected by routine clinical genotyping tests, might affect RFS even in stage IA, although ultra-low-level preT790M was thought not to have an impact on RFS in stage IA.

The above-mentioned study by Tatematsu et al. also did not show a significant effect of preT790M on OS in surgically resected *EGFR*-mutated NSCLC [36]. However, in the current study, multivariate analysis demonstrated that preT790M was the independent factor related to a poor prognosis in patients with resected *EGFR-*mutated NSCLC, irrespective of patient background including pathological stage, age, *EGFR* mutation subtype, and history of adjuvant chemotherapy. According to pathological stage, high-preT790M showed no prognostic impact in stage IA, but OS in high-preT790M tended to be shorter in stage IB or more advanced settings, as well as in the RFS analysis. To the best of our knowledge, the present research is the first to show that preT790M has a significant impact on OS in resected *EGFR*-mutated NSCLC in a larger cohort.

In metastatic stage settings, the appearance of T790M mutation after resistance to initial EGFR-TKI treatment (acquired T790M) has been reported to be associated with a good prognosis in the patients with *EGFR*-mutated NSCLC [22, 38, 39]. A basic research study found that the acquisition of T790M was associated with a slowing of tumor growth, which might underlie the good prognosis of *EGFR*-mutated NSCLC with acquired T790M [40]. On the other hand, positivity or high-abundance of preT790M has been demonstrated to be associated with poor efficacy of initial EGFR-TKI treatment or a poor prognosis [8–10, 12, 13, 16, 18, 19, 21, 22]. In the same way, the present study demonstrated that high-abundance of preT790M was correlated with poor RFS and OS in patients with early-stage NSCLC who had undergone surgical resection. These results suggest that clinical features are likely to be different between preT790M and acquired T790M [22]. However, the reason why patients with *EGFR*-mutated NSCLC harboring preT790M appear to have a poor prognosis has not yet been elucidated, even though the tumor harbors a low-level amount of T790M clones and has undergone surgical resection. The previous research showed that β-Catenin, which is involved in the pathogenesis and progression of malignant tumors, especially cancer stem cells, was upregulated and activated in *EGFR*-sensitizing mutant cells, and more in *EGFR*-mutant cells bearing T790M than in wild-type *EGFR* cells, and suggested that a cooperative association between β-catenin and *EGFR*-sensitizing mutations or with T790M plays a significant role in lung tumorigenesis [41]. Several studies also demonstrated that inhibition of β-catenin suppressed *EGFR*-activating and T790M mutated lung tumor growth or increased the anticancer effects of EGFR-TKIs [41, 42]. These data suggested that β-Catenin mediated stem-cell like properties of cancer cells harboring dual *EGFR*-activating mutation and T790M mutation may contribute to the activation of cell growth, proliferation, and the progression of disease; therefore, such research findings could explain the worse RFS in patients with NSCLC harboring concomitant preT790M. Further investigations and validation are needed.

Postoperative adjuvant chemotherapy is recommended for patients with completely resected stage II-IIIA and a subset of stage I NSCLC according to the results from large, randomized trials and meta-analyses that have demonstrated a significant OS benefit [43, 44]. However, whether driver mutation-positive patients with resected stage NSCLC also benefit from adjuvant chemotherapy had not been accurately clarified. In particular, for patients with resected NSCLC harboring *EGFR* mutations, given the role of EGFR-TKIs in advanced *EGFR-*mutant NSCLC, many clinical trials have been conducted to investigate the efficacy of EGFR-TKIs in the adjuvant setting [45–50]. Most trials demonstrated that adjuvant treatment using first-generation EGFR-TKIs can decrease the risk of recurrence and prolong DFS compared to placebo or chemotherapy, but these DFS advantages did not always translate to OS [51, 52]. A meta-analysis that evaluated the role of EGFR-TKIs as an adjuvant therapy for patients with completely resected *EGFR*-mutated NSCLC demonstrated that, compared to mono chemotherapy, early-generation EGFR-TKI monotherapy had a superior DFS benefit, but did not show a significant OS benefit, whereas treatment with EGFR-TKIs plus chemotherapy was associated with significantly longer DFS and OS compared to mono chemotherapy [53]. Therefore, these data suggested that it was necessary for the prolongation of DFS and OS in patients with resected *EGFR-*mutated NSCLC not only to add EGFR-TKIs as adjuvant treatment, but also to perform standard adjuvant chemotherapy as much as possible. On multivariate analysis in the current study, it was observed that OS in the patients who received adjuvant chemotherapy tended to be better than in those who did not receive adjuvant chemotherapy.

The ADAURA trial demonstrated significant improvement of DFS in the adjuvant osimertinib arm (HR for disease recurrence or death, 0.17 [99% CI, 0.11–0.26] in patients with stage II to IIIA disease, and 0.20 [99% CI, 0.14–0.30] in patients with stage IB to IIIA disease) [20]. In that trial, administration of standard postoperative adjuvant chemotherapy was allowed, but not mandatory, although the DFS benefit from osimertinib was documented regardless of whether patients undergone adjuvant chemotherapy. Based on the present study findings, high-preT790M was an independent factor related to a poor prognosis for both RFS and OS in stage IB or more advanced stages. In advanced settings, the AZENT study (NCT02841579) showed promising results for first-line osimertinib for patients with *EGFR*-mutated NSCLC with a coexisting low allelic fraction of T790M, in which the objective response rate was 77.3% and the median PFS was 23.1 months [54]. Moreover, the WJOG13119L study demonstrated that the time to treatment failure (TTF) of the micro-T790M-positive group treated by the first-generation EGFR-TKIs was shorter than the negative group, although the TTF of the micro-T790M-positive group treated by osimertinib was longer than that of the negative group [55]. Based on these reports, osimertinib may be expected to be effective for *EGFR*-mutated NSCLC with a low-frequency preT790M. Although the basic correlation between poor prognosis and minor-frequency preT790M in resected *EGFR-*mutated NSCLC has not yet been elucidated, osimertinib treatment may have had an impact on DFS for the population harboring potential preT790M in the ADAURA trial. Therefore, the addition of adjuvant osimertinib to standard adjuvant chemotherapy might also be expected to have a greater impact on improving OS. The NeoADAURA (NCT04351555) trial, investigating the efficacy and safety of neoadjuvant osimertinib in patients with *EGFR-*mutated resectable NSCLC, is ongoing. If translational research assessing minor-frequency T790M before osimertinib and after surgery could be conducted, the clinical significance of treatment of preT790M might be elucidated.

The limitations of the current study include the relatively short observation period and the low number of recurrence and death events, which results in a lack of statistical power, although the current study was the largest prospective trial, and the prognostic information was collected exactly. The number of patients in Stage I, especially stage IA, who have good prognosis because of the progress of diagnostic techniques and developments in improved surgical techniques was considerably large, which resulted in decreased incidences of recurrence and death. Therefore, further validation in a larger cohort might be needed for analysis of the prognosis for stage IB or more advanced settings. In stage IA cases, tumors mainly composed of ground glass opacity (GGO) components are often identified. The proportion of GGO tumors may have the potential to be related to why no difference between the high-preT790M group and the low-preT790M group was observed in RFS and OS in stage IA patients. However, information about the proportion of GGO tumor included in stage IA cases in the current study is not available.

There were several other limitations in the present study. In several previous analyses of prognostic factors for resected NSCLC, various covariates regarding postoperative pathological findings, such as lymphatic infiltration, vascular infiltration, and pleural invasion, were shown to be associated with a poor RFS or OS [25, 26]. However, the information regarding them was not collected in the JME study, and it remains unclear whether the factors of postoperative pathological findings affected prognosis in the present study. There was no provision regarding the CT scan interval in the JME study; it was left to the discretion of the attending physician, and, therefore, it may have affected RFS. Although the tumor specimens were dissected by pathologists, and those with as high a tumor content and as low a necrotic component as possible were chosen for analysis, the effect of normal cells could not be eliminated completely, resulting in a possible effect on accurate calculation of the preT790M-MAF.

## Conclusions

The current prospective, multicenter, observational study showed that a higher mutant-allele frequency of pretreatment T790M in patients with surgically resected *EGFR-*mutated NSCLC was associated with poorer RFS, independent of male sex, advanced pathological stage, and older age, and was also associated with worse OS, independent of *EGFR* mutation genotype, advanced pathological stage, older age, and no adjuvant chemotherapy.

## Supplementary Information

**Additional file 1.**

## Data Availability

The datasets generated during and/or analyzed during the current study are available from the corresponding author on reasonable request.

## Abbreviations

NSCLC: Non-small cell lung cancer
EGFR: Epidermal growth factor receptor
19del: Exon 19 deletions
L858R: Leu858Arg point mutations in exon 21
TKI: Tyrosine kinase inhibitor
preT790M: Pretreatment T790M
ddPCR: Droplet digital polymerase chain reaction
DFS: Disease-free survival
MAF: Mutant-allele frequency
RFS: Recurrence-free survival
OS: Overall survival
HR: Hazard ratio
CI: Confidence interval
