# Supplementary material for: Prognostic impact of pretreatment T790M mutation on outcomes for patients with resected, EGFR-mutated, non-small cell lung cancer
Source: BMC Cancer. 2022 Jul 15;22:775. doi: 10.1186/s12885-022-09869-7 (PMC9288048; doi:10.1186/s12885-022-09869-7)
Supplement: Supplementary file 1 — Additional file 1. [file 12885_2022_9869_MOESM1_ESM.docx]

**Supplementary Fig. S1.**

**
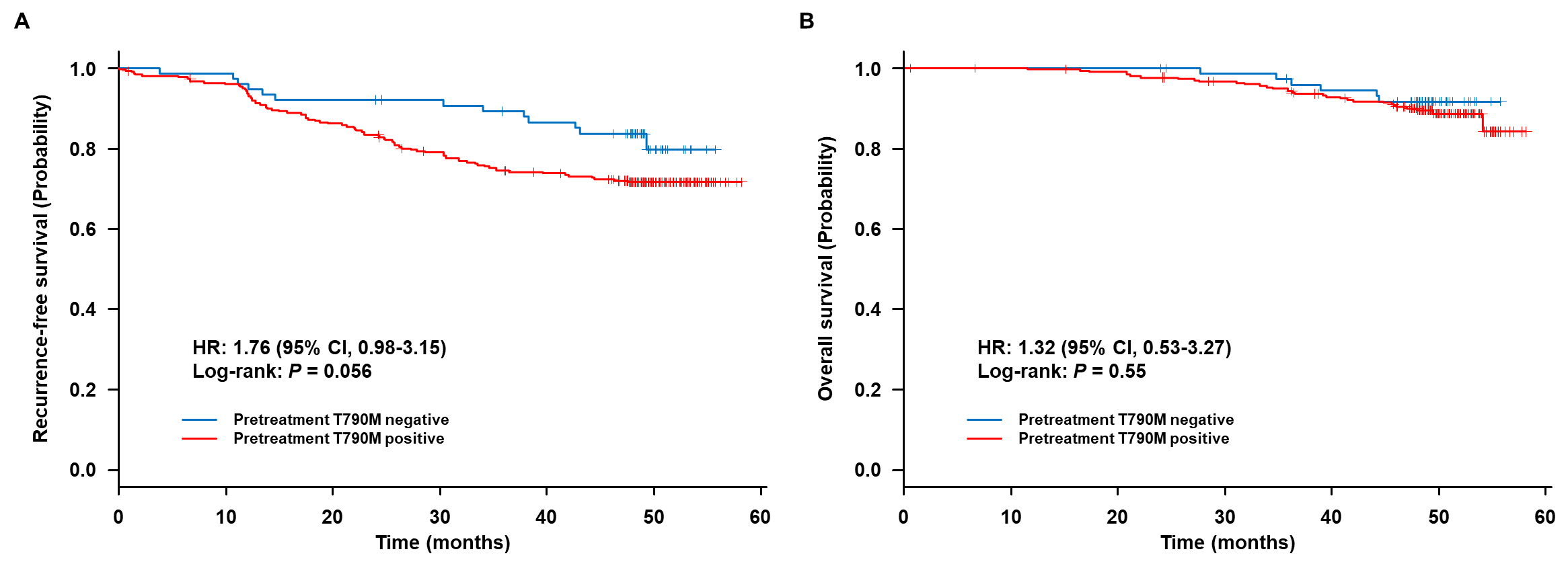
**

**Supplementary Fig. S1.** Kaplan-Meier curves for recurrence-free survival (A) and overall survival (B) according to pretreatment T790M status (positive vs negative). Plus signs denote censoring.

**Supplementary Fig. S2.**

**
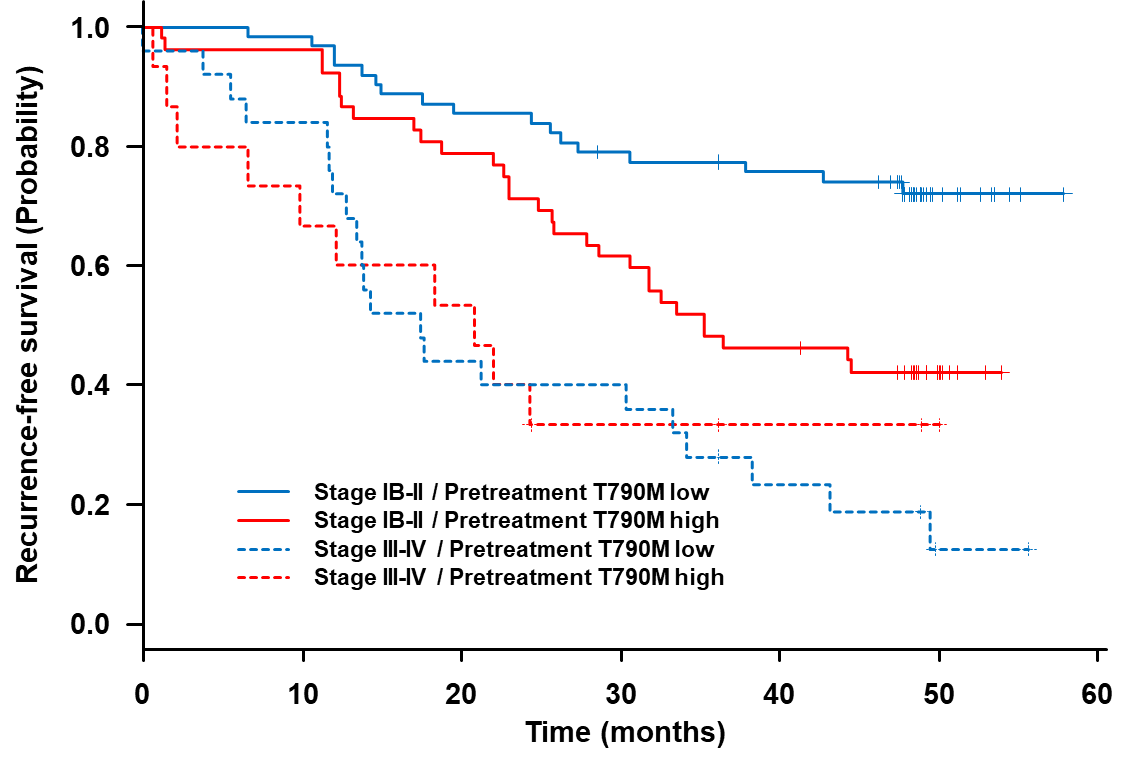
**

**Supplementary Fig. S2.** Kaplan-Meier curves for recurrence-free survival according to pretreatment T790M status (high vs low) and pathological stage (IB-II vs III-IV). Plus signs denote censoring.

**Supplementary Fig. S3.**


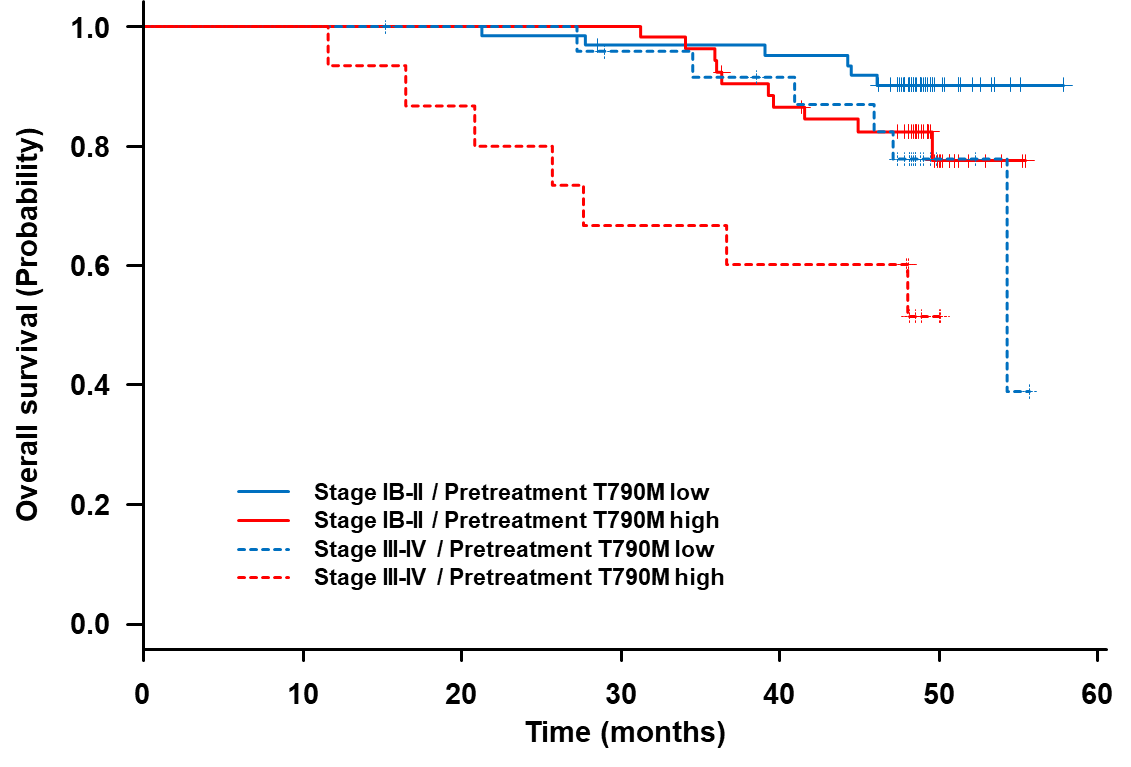


**Supplementary Fig. S3.** Kaplan-Meier curves for overall survival according to pretreatment T790M status (high vs low) and pathological stage (IB-II vs III-IV). Plus signs denote censoring.
